# Melittin suppresses growth and induces apoptosis of non-small-cell lung cancer cells via down-regulation of TGF-β-mediated ERK signal pathway

**DOI:** 10.1590/1414-431X20209017

**Published:** 2020-12-18

**Authors:** Renzhi Yu, Miao Wang, Minghuan Wang, Lei Han

**Affiliations:** 1 Department of Respiratory Medicine, Mudanjiang Medical University Affiliated Hongqi Hospital, Mudanjiang, China; 2 Community Health Service Center, Mudanjiang Medical University Affiliated Hongqi Hospital, Mudanjiang, China

**Keywords:** Melittin, NSCLC, Apoptosis, TGF-β, ERK

## Abstract

The purpose of this study was to investigate the anti-cancer effect of melittin on growth, migration, invasion, and apoptosis of non-small-cell lung cancer (NSCLC) cells. This study also explored the potential anti-cancer mechanism of melittin in NSCLC cells. The results demonstrated that melittin suppressed growth, migration, and invasion, and induced apoptosis of NSCLC cells *in vitro*. Melittin increased pro-apoptotic *caspase-3* and *Apaf-1* gene expression. Melittin inhibited tumor growth factor (TGF)-β expression and phosphorylated ERK/total ERK (pERK/tERK) in NSCLC cells. However, TGF-β overexpression (pTGF-β) abolished melittin-decreased TGF-β expression and pERK/tERK in NSCLC cells. Treatment with melittin suppressed tumor growth and prolonged mouse survival during the 120-day observation *in vivo*. Treatment with melittin increased TUNEL-positive cells and decreased expression levels of TGF-β and ERK in tumor tissue compared to the control group. In conclusion, the findings of this study indicated that melittin inhibited growth, migration, and invasion, and induced apoptosis of NSCLC cells through down-regulation of TGF-β-mediated ERK signaling pathway, suggesting melittin may be a promising anti-cancer agent for NSCLC therapy.

## Introduction

Lung cancer is the leading cause of cancer-related mortality in the world (1). The majority of newly diagnosed lung cancer cases are non-small-cell lung cancer (NSCLC), of which up to half are considered locally advanced at the time of diagnosis (2). A study showed that mortality of NSCLC is very high, with 5-year survival rates around 15-20% (3). Drug resistance is a major cause for therapeutic failure in NSCLC leading to most of the cancer-associated mortality, tumor recurrence, and disease progression (4–6).

Pathologically, NSCLC includes large cells carcinoma, squamous cells carcinoma, and adenocarcinoma and it is closely associated with distant metastasis (7–9). Owing to the lack of major advancements in treatment, the strategies for NSCLC patients therapy remains limited, and the 5-year overall survival rate is poor due to more than two thirds of lung cancer patients being diagnosed at an advanced stage (10–12). Therefore, more efficient anti-cancer therapies for NSCLC therapy are required to improve time to progression and 5-year overall survival.

Melittin is a major toxic component of bee venom (*Apis mellifera*), which has been shown to have anti-inflammatory and anti-nociceptive properties in cells and animal disease models (13). Melittin was reported to be involved in apoptosis of human carcinoma cells by activating CaMKII-TAK1-JNK/p38 and inhibiting IκBα kinase-NF-κB pathways (14). Evidence indicates that melittin is a potent cytolytic toxin for tumor cells via *in vitro*- and *in vivo*-targeted tumor lysis (15,16). In addition, melittin suppresses VEGF-A-induced tumor growth by blocking VEGFR-2 and the COX-2-mediated MAPK signaling pathway in lung cancer cells (17). Furthermore, Qin et al. (18) suggested that melittin inhibits tumor angiogenesis by modulating the SDF-1alpha/CXCR4 signaling pathway in a UMR-106 osteosarcoma xenograft mouse model. Zhang et al. (19) indicated that the antitumor activity of melittin is associated with the antiangiogenic actions of inhibiting the VEGF and hypoxia-inducible factor signaling pathways. Additionally, Gao et al. (20) found that melittin presented an anti-cancer effect for NSCLC via inducing apoptosis by regulation of miR-183 expression. Bee venom and its compounds such as melittin have antitumor, immunomodulatory, and apoptotic effects in different tumor cells *in vivo* and *in vitro* (21). However, it is unclear how melittin inhibits NSCLC growth, migration, and invasion *in vitro* and *in vivo*.

In this study, we investigated the antitumor effect of melittin in NSCLC cells and the NSCLC xenograft mouse model. We firstly evaluated the antitumor mechanism of melittin, and then assessed the association between melittin and tumor growth factor (TGF)-β-mediated ERK signal pathway in growth and apoptosis of NSCLC cells.

## Material and Methods

### Cell cultures

A549 and H358 cell lines were purchased from American Type Culture Collection. A549 cells are from a human lung carcinoma cell line and H358 cells are from human lung adenocarcinoma. A549 and H358 cells were cultured in RPMI 1640 medium (Thermo Fisher Scientific, USA) supplemented with 10% heat-inactivated fetal bovine serum (FBS, Thermo), 3 mM L-glutamine, 50 μg/mL gentamicin (Biowhittaker, USA), and 1% penicillin/streptomycin (Sigma, Germany). Cells were cultured at 37°C and 5% CO_2_. Cells were treated with melittin (2 μg/mL) for 24, 48, and 72 h for further analyses.

### Real-time quantitative PCR (RT-qPCR) analysis

Total RNA was extracted from A549 and H358 cells by RNAeasy Mini kit (QIAGEN, USA). Expression levels of caspase-3 and Apaf-1 in A549 and H358 cells were measured by RT-qPCR with β-actin as an endogenous control (22) (Invitrogen, USA). All forward and reverse primers were synthesized by Invitrogen [caspase-3: 5′-GCCAGACTACATGGAAATCTA-3′ (forward), 5′-GCAAGGACAAGATTCGATACT-3′ (reverse); Apaf-1: 5′-CTTCTCACTGTCGACTACCGC-3′ (forward), 5′-GCGTCTCCTGTGCATTCG-3′ (reverse)]. Relative mRNA expression changes were calculated by 2^−ΔΔCt^ (23). The results are reported as the n-fold compared to β-actin.

### Overexpression of TGF-&mac_bgr;

*TGF-β* gene (sense, 5′-AACTGCTCAACACCGGAATTT-3′; antisense, 5′-CTGTATTCCGTCTCCTTGGTTC-3′) was cloned into pCMVp-NEO (Addgene, USA) with β-actin (sense, 5′-CCTTCCGTGTTCCTACCCC-3′; antisense, 5′-GCCCAAGATGCCCTTCAGT-3′) as control. The recombinants were named pCMVp-NEO-TGF-β (100 pmol, pTGF-β) or pCMVp-NEO-control (pControl). A549 (1×10^5^ cells/well) and H358 (1×10^5^ cells/well) cells were cultured in six-well plates until 90% confluence. The medium was then removed and cells were transfected by pCMVp-NEO-TGF-β (50 nM) or pCMVp-NEO (50 nM, pControl) using Lipofectamine 2000 (Sigma-Aldrich, Germany) according to the manufacturer protocols. Expression of TGF-β in A549 and H358 cells was used for further analysis after 72 h transfection. Cells successfully TGF-β-transfected were selected using G418 (600 mg/L) screening for 4 times as described previously (24).

### MTT cytotoxicity assays

A549 and H358 cells were incubated with melittin (1.0, 1.5, 2.0, and 2.5 μg/mL) in 96-well plates for 24, 48, and 72 h in triplicate for each condition, and PBS was added instead of melittin as a control. At each time point, 20 μL of MTT (5 mg/mL) in PBS solution was added to each well, and the plate was further incubated for 4 h. Most of the medium was removed and 100 μL of DMSO (dimethyl sulfoxide) was added to the wells to solubilize the crystals. The absorbance was measured by a BIO-RAD (ELISA) reader (USA) at wavelength of 540 nm.

### Cell invasion and migration assays

Cell invasion and migration assays were performed as described previously (25). In brief, A549 and H358 cells were treated with melittin (2 μg/mL) for 24 h and non-treated cells were used as control. Migration and invasion of A549 and H358 cells was conducted in a 6-well culture plate with chamber inserts (BD Biosciences, USA). For migration assays, 1×10^4^/well concentration of the A549 and H358 cells were placed into the upper chamber with the non-coated membrane for 48 h at 37°C. For invasion assays, cells (1×10^4^/well) were placed into the upper chamber with the Matrigel-coated membrane for 48 h at 37°C. Cells were fixed in 4% paraformaldehyde at 25°C for 15 min and stained with 0.1% crystal violet dye (Sigma-Aldrich) at 25°C for 15 min. Migration and invasion of A549 and H358 cells were counted in at least three random fields of every membrane in a microscope (magnification ×50; Olympus, Japan). The percentage of migrated cells was qualified as follows: (control - melittin) / control × 100%.

### Flow cytometry analysis

A549 and H358 cells were cultured until 90% confluence was reached. Apoptosis was assessed after H358 cells were incubated with melittin (2 μg/mL) for 48 h. A549 and H358 cells were trypsinized and collected after incubation. The cells were then washed in cold PBS, adjusted to 1×10^6^ cells/mL with PBS, labeled with annexin V-FITC and PI (BD, USA), and analyzed with a FACScan flow cytometer (BD). Quantitative analysis of apoptotic cells was carried out using BD FACSuite software v2.0 (26).

### Western blot analysis

A549 (1×10^7^) and H358 (1×10^7^) cells were lysed in RIPA buffer (Sigma-Aldrich) followed by homogenization at 4°C for 10 min. Protein concentrations were measured using BCA Protein assay kit (Thermo Fisher Scientific). Proteins (30 μg) were analyzed by 12% SDS-PAGE assays followed by transfer to PVDF membranes. Proteins were incubated with rabbit anti-human ERK1/2 (1:500, ab93125, Abcam, China), pERK (1:1,500, phospho-Thr202/Tyr204, 1:1,000, ab214362, Abcam), TGF-β (1:1,500, ab31013, Abcam), and β-actin (1:1,500, ab8226, Abcam) for 12 h at 4°C. Membranes were incubated with HRP-conjugated goat anti-rabbit IgG (cat. No. 4410; Cell Signaling Technology, USA; 1:2,000) secondary antibodies at 37°C for 2 h. Immunoreactivity was evaluated using ECL western blotting kit (Beyotime Institute of Biotechnology, China). Quantitation of signal intensities was evaluated using UVP EC3 v3.0 software (UVP, LLC, USA).

### Animal study

A total of 48 specific pathogen-free (SPF) male nude mice (six weeks old, 28-35 g body weight) were purchased from Northeast Agricultural University (Harbin, China). Mice were housed at 23±0.5°C and humidity of 50±5%, with a 12-h light/dark cycle and free access to food and water. Mice were subcutaneously implanted with A549 or H358 cells (1×10^6^) into the right flank and divided into four groups (n=12 in each group). Treatments started on day 6 after tumor implantation when the tumor diameter reached 5-8 mm. Mice received a local injection of melittin (5 mg/kg) or PBS, once every 3 days for a total of 21 days. The tumor volumes were calculated according to a previous study (27). On day 22, mice were sacrificed (n=3 per group) using cervical decapitation under *iv* pentobarbital (35 mg/kg), and tumors were obtained for further analyses. The remaining animals were used for long-term (120 days) observations without any treatment. No animal presented multiple subcutaneous tumors. During the long-term experiment, tumor volume and animal health were monitored every three days. Mice were sacrificed using decapitation when tumor diameter reached 10 mm (tumor volume: <523.33 mm^3^) during the 120-day experiment.

### Immunohistochemistry

Tumor tissues from xenograph mice after treatment with melittin or PBS were fixed with 10% formaldehyde, embedded in paraffin, and cut into serial sections of 4-μm thickness. Tumor samples were cut into 4-μm tumor sections and antigen retrieval was performed using 0.01 M citrate buffer (pH 6.0) for 40 min at 90°C. Tumor sections were incubated with rabbit anti-human ERK (1:1,500, ab184699, Abcam) and TGF-β (1:1,500, ab31013, Abcam). Then, tumor tissues were incubated using secondary antibody (Alexa Fluor 488) (1: 2,000, Jackson, Abcam) at 37°C for 2 h. The result was captured at 100× magnifications using the Olympus IX73 microscope. Expression levels of proteins were quantified using Image Pro Plus software (version 5.0, Media Cybernetics, Inc., USA).

### TUNEL assay

Terminal deoxynucleotidyl transferase-mediated biotinylated UTP nick end labeling (TUNEL) staining was used to analyze apoptotic cells in NSCLC tumor tissues using ApopTag kit (Millipore, USA) according to the manufacturer's instructions. Briefly, paraffin tumor sections were fixed with 4% paraformaldehyde for 5 min at 37°C followed by permeabilization with 0.1% Triton X-100. Tissue sections were then incubated with TUNEL reaction mixture at 37°C for 30 min as described previously (28). The sections of tumor were colored with diaminobenzidine at 37°C for 15 min followed by staining with hematoxylin at 37°C for 10 min. The percentage of TUNEL-positive cells was examined and determined by the apoptotic cells ratio in six randomly selected fields using a confocal laser scanning microscope (FV300, Olympus). Statistical quantification of TUNEL-positive tumor cells was calculated to analyze the efficacy of melittin for inhibition of tumor growth. The staining intensity of the TUNEL-positive cells was quantified using the software Image-Pro Plus^®^ (Media Cybernetics, Inc., USA).

### Statistical methods

All data are reported as means±SE. Each experiment was repeated as least three times. Unpaired data were compared by Student's *t*-test and comparisons of multiple groups were done by analysis of variance (ANOVA) followed by Tukey's *post hoc* test. Kaplan-Meier was used to estimate the risk of melittin treatment during 120-day treatment and analyzed using log-rank test. *P<0.05 was considered statistically significant.

## Results

### Melittin inhibited growth, migration, and invasion of NSCLC cells

**Figure 1:**
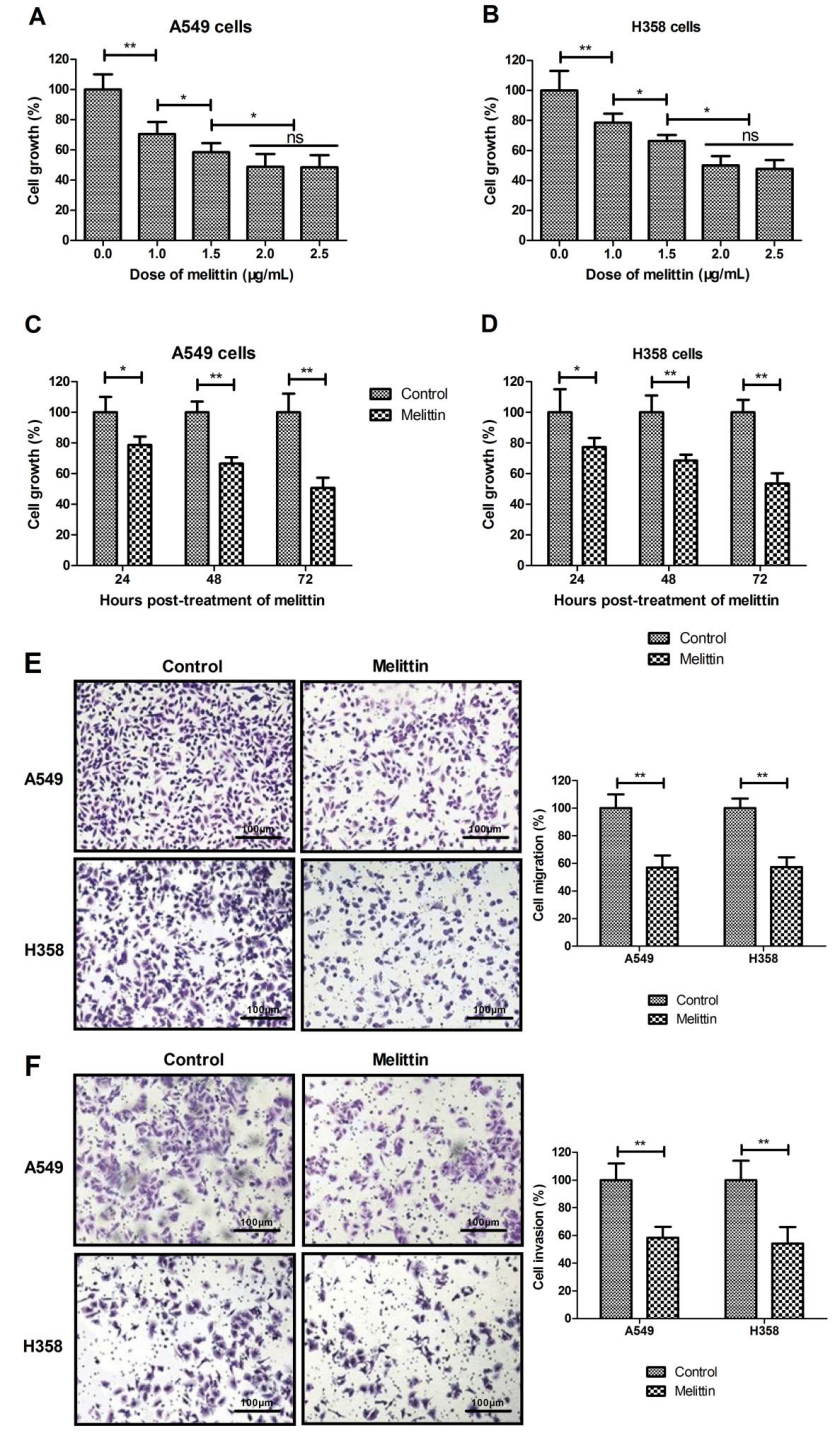
Melittin inhibited growth, migration, and invasion of non-small-cell lung cancer (A549 and H358) cells *in vitro.* **A** and **B**, Effects of different doses of melittin on cell growth after 72-h incubation. **C** and **D**, Melittin (2.0 μg/mL) treatment suppressed cell growth in a time-dependent manner (24, 48, and 72 h). Control, PBS-treated cells. **E** and **F**, Melittin (2.0 μg/mL) inhibited migration (**E**) and invasion (**F**) of cells (scale bar: 100 μm). *P<0.05, **P<0.01 (Student's *t*-test and ANOVA). ns: not significant.

As shown in Figure 1A and B, melittin suppressed A549 and H358 cells growth in a dose-dependent manner compared to control. As illustrated in Figure 1C and D, melittin presented time-dependent inhibitory effect compared to control. Results demonstrated that 2.0 mg/mL of melittin maximally inhibited cell growth compared to control. Migration and invasion assays showed that melittin (2.0 mg/mL) markedly inhibited migration and invasion of A549 and H358 cells (Figure 1E and F). These results suggested that melittin could inhibit NSCLC cells growth, migration, and invasion.

### Melittin promoted apoptosis of NSCLC cells

**Figure 2:**
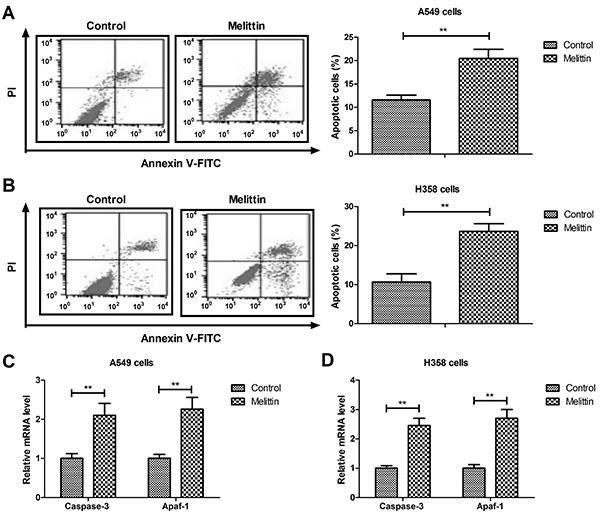
Melittin induced apoptosis of non-small-cell lung cancer cells (A549 and H358). **A** and **B**, Effects of melittin (2.0 μg/mL) on apoptosis. **C** and **D**, Effects of melittin on pro-apoptosis gene expression of *caspase-3* and *Apaf-1*. **P<0.01 (Student's *t*-test).

As shown in Figure 2A and B, melittin induced A549 and H358 cells apoptosis compared to control. Results demonstrated that melittin increased mRNA expression of pro-apoptotic genes *caspase-3* and *Apaf-1* (Figure 2C and D). These results showed that melittin could induce apoptosis of NSCLC cells through increasing gene expression of *caspase-3* and *Apaf-1*.

### Melittin inhibited growth of NSCLC cells through TGF-&mac_bgr;-mediated ERK signaling pathway

**Figure 3:**
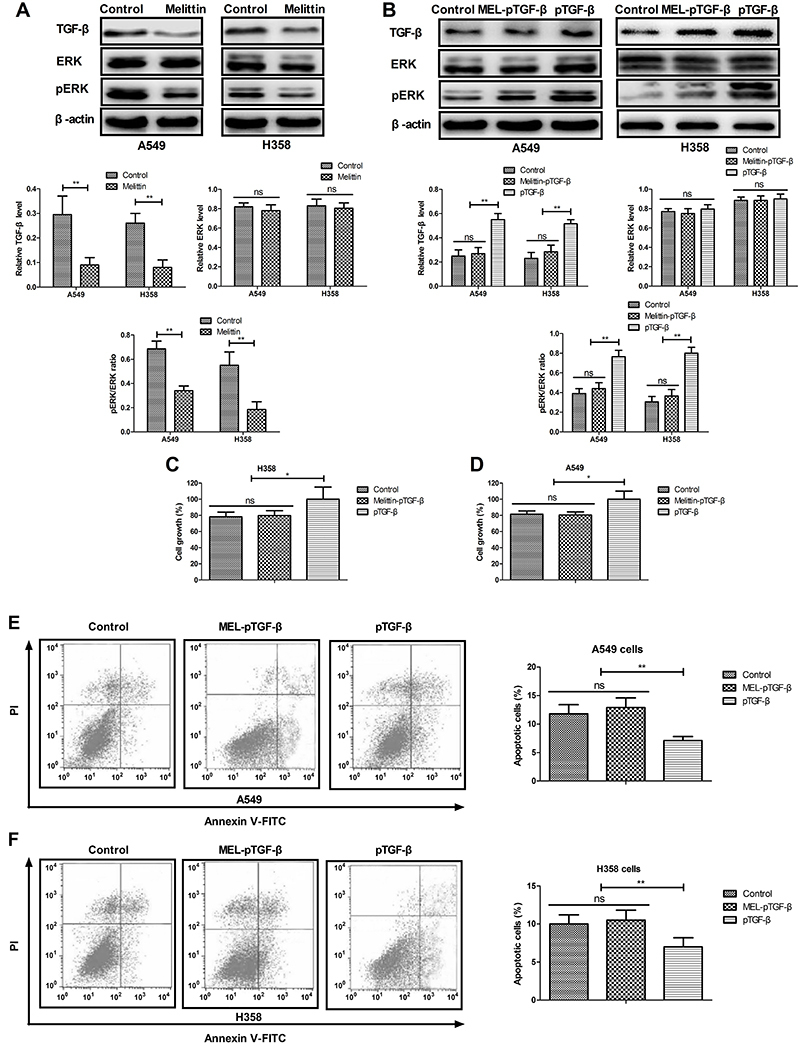
Melittin inhibited growth of non-small-cell lung cancer cells (A549 and H358) via tumor growth factor (TGF)-β-mediated ERK signaling pathway. **A**, Melittin (2.0 μg/mL) downregulated TGF-β, ERK, and pERK expression. **B**, TGF-β overexpression (pTGF-β) canceled melittin-inhibited TGF-β expression and phosphorylation levels of ERK. **C** and **D**, TGF-β overexpression (pTGF-β) abolished melittin-inhibited (MEL-pTGF-β) growth. **E** and **F**, Effects of TGF-β overexpression (pTGF-β) on melittin-induced apoptosis. *P<0.05, **P<0.01 (Student's *t*-test and ANOVA). ns: not significant.

As shown in Figure 3A, melittin decreased TGF-β expression and phosphorylated ERK/total ERK levels in A549 and H358 cells. Results demonstrated that TGF-β overexpression (pTGF-β) canceled melittin-inhibited (MEL-pTGF-β) TGF-β expression, and phosphorylation ERK/total ERK (pERK/tERK) in cells (Figure 3B). TGF-β overexpression (pTGF-β) abolished melittin-inhibited growth of A549 and H358 cells (Figure 3C and D). We observed that TGF-β overexpression abolished melittin-induced apoptosis of A549 and H358 cells (Figure 3E and F). These results suggested that melittin administration inhibits NSCLC growth through TGF-β-mediated ERK signaling pathway.

### *In vivo* effects of melittin on NSCLC xenograft mouse model

**Figure 4:**
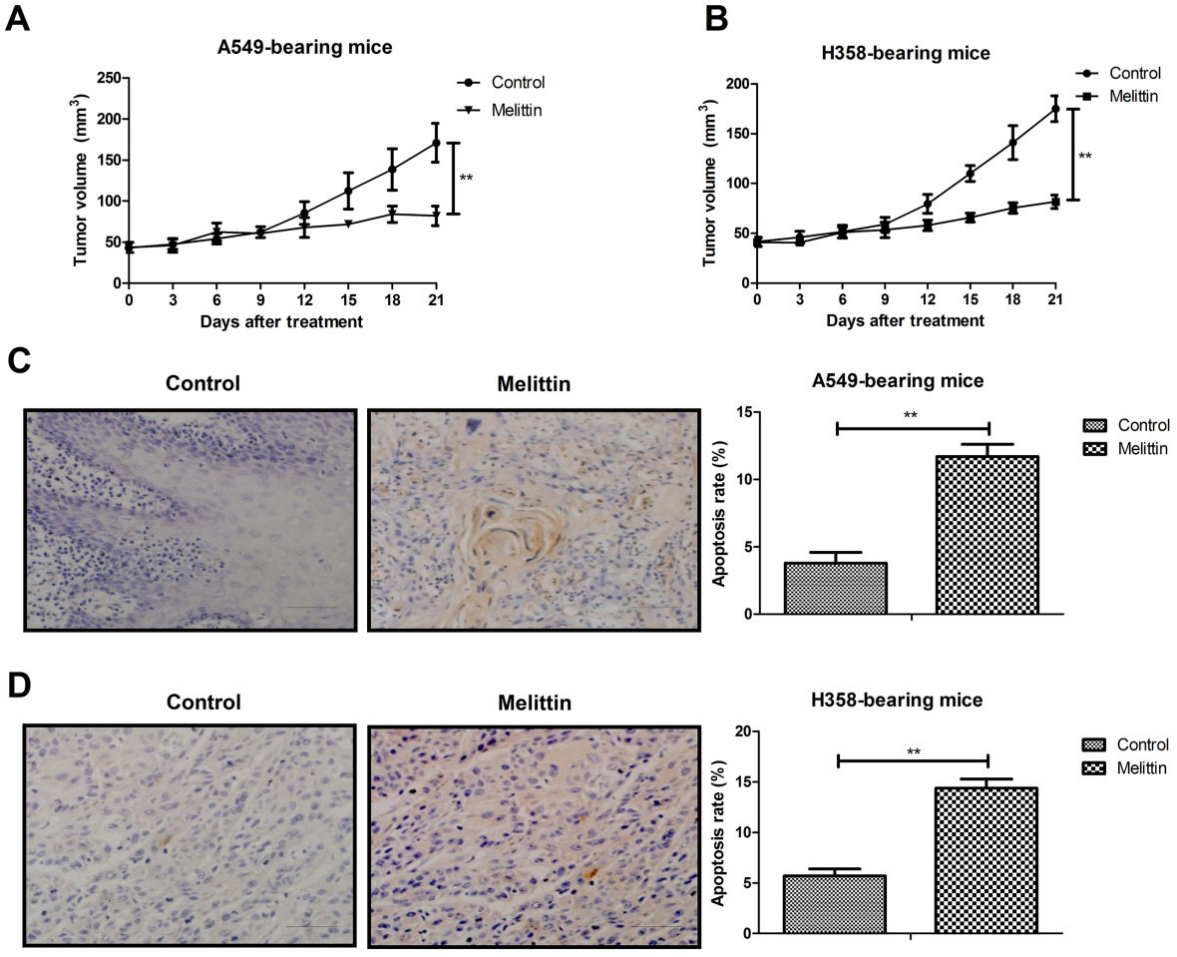
*In vivo* efficacy of melittin for non-small-cell lung cancer cells (A549 and H358) in a xenograft mouse model. **A** and **B**, Melittin (5 mg/kg) administration inhibited tumor growth. **C** and **D**, Melittin administration increased percent of apoptosis in tumor tissues (scale bar: 100 μm). **P<0.01 (Student's *t*-test).

**Figure 5:**
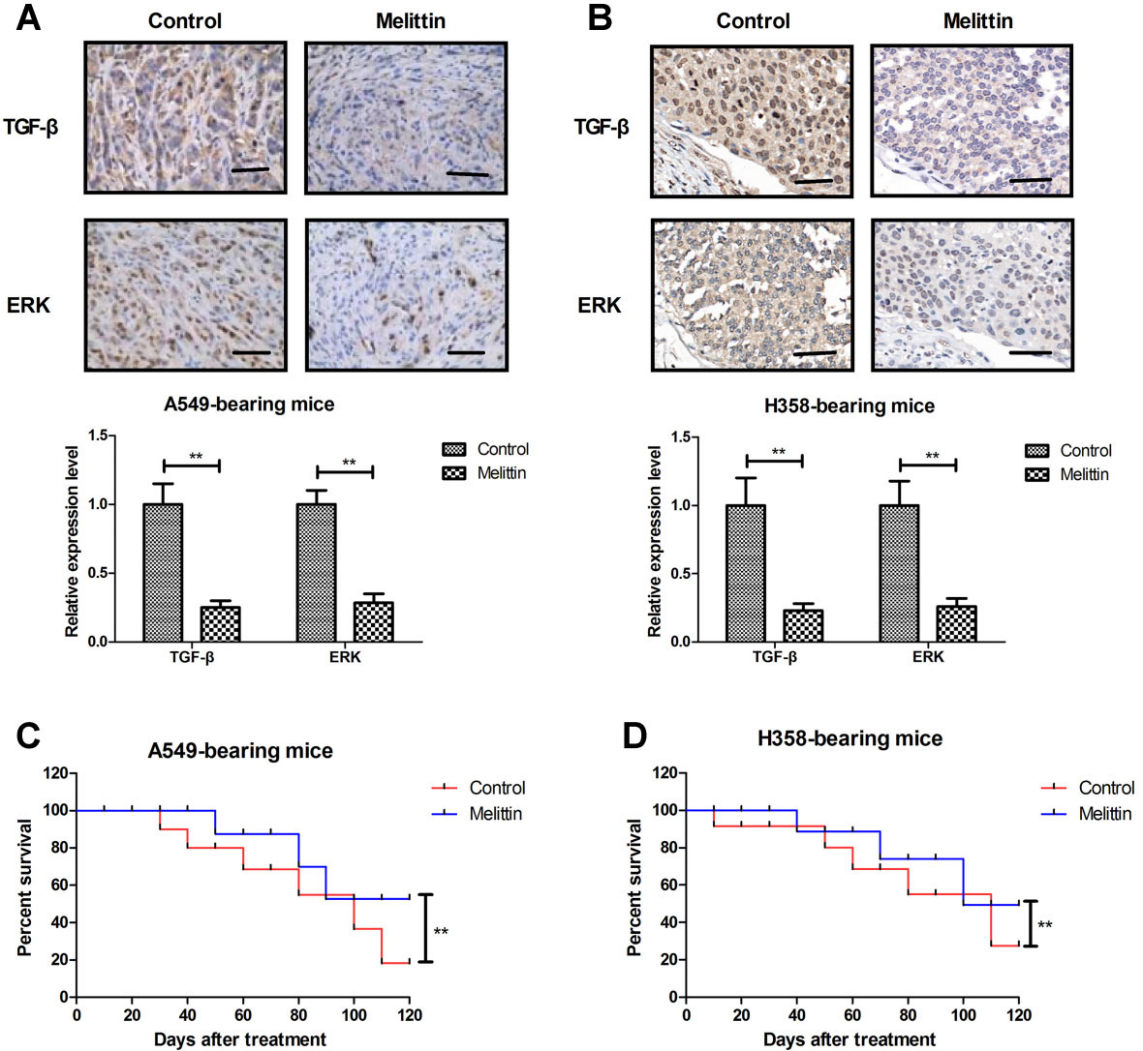
Aand **B**, Effects of melittin on expression levels of tumor growth factor (TGF)-β and ERK in tumor tissues (scale bar: 50 μm). **C** and **D**, Melittin administration prolonged animal survival compared to PBS-treated mice in a 120-day observation. **P<0.01 (Student's *t*-test).

**Figure 6:**
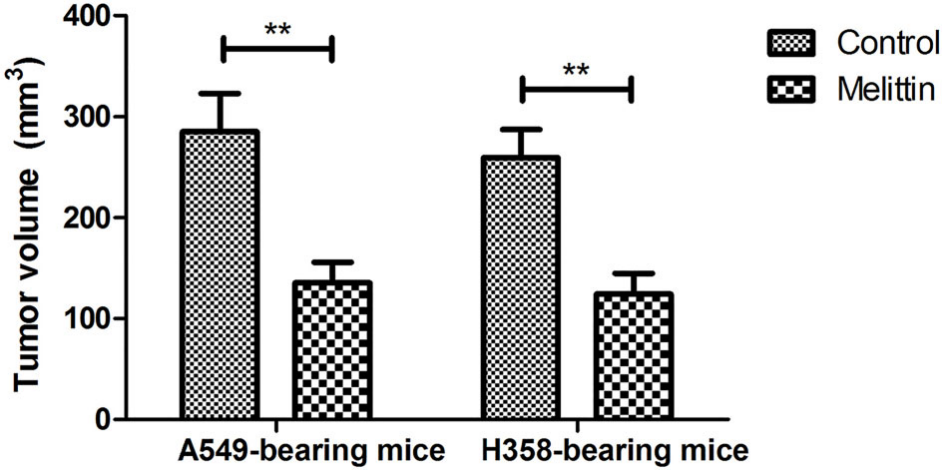
Tumor volume in experimental mice on day 120. **P<0.01 (Student's *t*-test).

Melittin administration inhibited tumor growth compared to PBS-treated mice (Figure 4A and B). TUNEL assay demonstrated that apoptotic cells were increased by melittin treatment in A549- and H358-bearing mice (Figure 4C and D). Expression levels of TGF-β and ERK were down-regulated in melittin-treated tumor compared to PBS (Figure 5A and B). Melittin administration prolonged animal survival in a 120-day observation (Figure 5C and D). The tumor volume of experimental mice on day 120 is shown in Figure 6. These results suggested that melittin is an efficient anti-cancer agent for NSCLC *in vivo*.

## Discussion

Despite more and more therapeutic options being developed for NSCLC patients, the survival rate of patients is poor, which is a critical clinical problem (29,30). Evidence shows that melittin could suppress invasion and angiogenesis via blocking of the VEGF-A/VEGFR-2/MEK1/ERK1/2 pathway in human hepatocellular carcinoma (31). Importantly, melittin treatment led to suppression of HIF-1alpha/VEGF expression through inhibition of ERK signal pathway in human cervical carcinoma cells (32). However, these findings still cannot explain the mechanism of action of melittin. Findings in the current study indicated that melittin administration significantly inhibited NSCLC cells migration and invasion, as well as induced apoptosis through TGF-β-mediated ERK signal pathway.

Tumor apoptosis is a hallmark in the pathogenesis and treatment for human cancer patients (33–35). Li et al. (36) indicated that growth arrest and apoptosis of the human hepatocellular carcinoma cell line BEL-7402 could be induced by melittin by up-regulation of Fas expression. In addition, melittin inhibited the proliferation of MG63 cells by activating inositol-requiring protein-1alpha and X-box binding protein 1-mediated apoptosis (37). Results in this study indicated that melittin administration induced apoptosis of NSCLC cells and increased pro-apoptosis gene *caspase-3* and *Apaf-1*. Furthermore, melittin radiosensitized esophageal squamous cell carcinoma with induction of apoptosis *in vitro* and *in vivo*, indicating that melittin may be a potentially promising radiosensitizer in esophageal squamous cell carcinoma radiation therapy (38). This implied that melittin induced cell apoptosis associated with induction of the caspase-3-dependent endoplasmic reticulum apoptosis pathway. However, further studies are required to elucidate the apoptotic mechanisms of melittin in NSCLC cells.

TGF-β1-induced epithelial-to-mesenchymal transition pathway is associated with lung cancer progression, which can further contribute to growth of NSCLC (39). Data in the current study showed that melittin inhibited TGF-β expression in NSCLC cells and tumor tissue. Importantly, melittin treatment prolonged the survival of tumor-bearing mice by inducing apoptosis. Immunohistochemistry demonstrated that expression levels of TGF-β and phosphorylation ERK levels were also down-regulated in melittin-treated tumors, leading to apoptosis of tumor cells. Therefore, up-regulation of TGF-β and phosphorylation ERK levels may be two contributors to the development of NSCLC.

In summary, our results suggested that melittin played an important role in the progression of NSCLC. Full understanding of the inhibitory role of melittin in NSCLC growth might provide a novel therapeutic strategy by suppressing TGF-β-mediated ERK signal pathway. However, further research on the therapeutic effects of melittin is required to evaluate the inhibitory effects and molecular mechanism in other human tumors.
